# Attenuation of Chronic Inflammation in Intestinal Organoids with Graphene Oxide-Mediated Tumor Necrosis Factor-α_Small Interfering RNA Delivery

**DOI:** 10.1021/acs.langmuir.3c02741

**Published:** 2024-02-07

**Authors:** Sadman Sakib, Shan Zou

**Affiliations:** Metrology Research Centre, National Research Council of Canada, 100 Sussex Drive, Ottawa, ONK1A 0R6, Canada

## Abstract

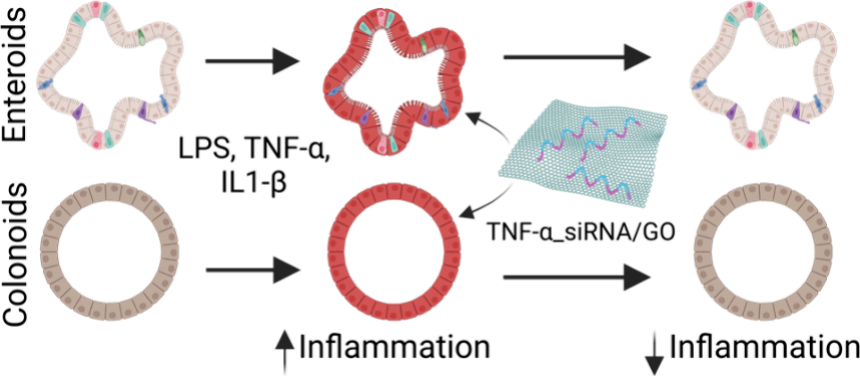

Inflammatory bowel disease (IBD) is a chronic inflammatory disease of the gastrointestinal tract with a complex and multifactorial etiology, making it challenging to treat. While recent advances in immunomodulatory biologics, such as antitumor necrosis factor-α (TNF-α) antibodies, have shown moderate success, systemic administration of antibody therapeutics may lead to several adverse effects, including the risk of autoimmune disorders due to systemic cytokine depletion. Transient RNA interference using exogenous short interfering RNA (siRNA) to regulate target gene expression at the transcript level offers an alternative to systemic immunomodulation. However, siRNAs are susceptible to premature degradation and have poor cellular uptake. Graphene oxide (GO) nanoparticles have been shown to be effective nanocarriers for biologics due to their reduced cytotoxicity and enhanced bioavailability. In this study, we evaluate the therapeutic efficacy of GO mediated TNF-α_siRNA using in vitro models of chronic inflammation generated by treating murine small intestines (enteroids) and large intestines (colonoids) with inflammatory agents IL-1β, TNF-α, and LPS. The organotypic mouse enteroids and colonoids developed an inflammatory phenotype similar to that of IBD, characterized by impaired epithelial homeostasis and an increased production of inflammatory cytokines such as TNF-α, IL-1β, and IL-6. We assessed siRNA delivery to these inflamed organoids using three different GO formulations. Out of the three, small-sized GO with polymer and dendrimer modifications (smGO) demonstrated the highest transfection efficiency, which led to the downregulation of inflammatory cytokines, indicating an attenuation of the inflammatory phenotype. Moreover, the transfection efficiency and inflammation-ameliorating effects could be further enhanced by increasing the TNF-α_siRNA/smGO ratio from 1:1 to 3:1. Overall, the results of this study demonstrate that ex vivo organoids with disease-specific phenotypes are invaluable models for assessing the therapeutic potential of nanocarrier-mediated drug and biologic delivery systems.

## Introduction

Inflammation is a critical response of the immune system to infection or injury, serving to repair and restore tissue homeostasis. While acute inflammation generally protects against harmful stimuli, prolonged and chronic inflammation can have detrimental effects and disrupt tissue homeostasis. A prominent example of chronic inflammation is inflammatory bowel disease (IBD), which encompasses Crohn’s disease, primarily affecting the small intestines, and ulcerative colitis, typically found in the large intestines or colon tissue.^1^ IBD patients suffer from chronic inflammation of the gut and are at a higher risk of developing colorectal cancer.^2^ The etiopathology of IBD remains elusive but is generally believed to result from genetic predispositions, dysregulation of immune responses, and loss of homeostasis in the gastrointestinal microbiota.^3^ Due to the complex and multifactorial etiology of IBD, effective treatment remains elusive. Conventional therapeutics such as 5-aminosalicylic acid derivatives, glucocorticoids, and immunosuppressants have achieved limited success, often leading to reduced therapeutic efficacy, adverse effects, and dependency.^2,4^

IBD is characterized by the dysregulation of inflammatory cytokines, including tumor necrosis factor-α (TNF-α), interferon-γ (IFN-γ), interleukin-4 (IL-4), interleukin-10 (IL-10), and interleukin-21 (IL-21).^5−7^ Targeting these key inflammatory cytokines or their downstream effectors has become a viable avenue for managing inflammation.^7,8^ Targeting IFN-γ with fontolizumab, a humanized anti-IFN-γ antibody, or silencing its downstream target, Janus kinase-1 (JAK1) and Janus kinase-3 (JAK-3), has shown transient moderate attenuation of inflammation.^8,9^ Another major target for the management of inflammation is the cytokine TNF-α, which plays a significant role in immune responses in IBD,^7^ being widely expressed by all the inflamed tissues.^5,6^ Moreover, TNF-α is responsible for the release of other inflammatory cytokines such as interleukin-8 (IL-8), interleukin-6 (IL-6), and matrix metalloproteinases (MMPs),^5^ as well as chemokines that recruit neutrophils to inflammatory sites, thus propagating inflammation.^10^ Anti-TNF-α antibodies, such as infliximab, adalimumab, and golimumab, have been successfully implemented in the treatment of IBD.^7,11^ However, antibody therapeutic modalities have limitations, including the risk of infections, demyelinating diseases, and induction of autoimmune diseases due to systemic cytokine depletion.^7,12^

A viable alternative to systemic antibody administration is the gene silencing of target inflammatory cytokines via transient small interfering RNAs (siRNAs). Gene silencing of TNF-α has shown downregulation of MMPs and a reduction in paw swelling and joint destruction in rheumatoid arthritis mouse models.^6^ Furthermore, TNF-α_siRNA in mouse models of alveolar inflammation reduced IL-6 and interleukin 1β (IL-1β) secretion and decreased eosinophil infiltration.^13^ Similar effects have been found in acute models of colitis.^14^ However, gene therapeutics are susceptible to premature degradation and poor cellular uptake due to their hydrophilic nature and cationic charge, limiting their efficacy.^15−17^ Effective delivery mechanisms for nucleic acids are needed to enhance the bioavailability and therapeutic benefits.

In recent years, nanocarrier mediated delivery of drugs and biologics has garnered widespread interest.^18^ Graphene oxide (GO) is a single-atomic layered two-dimensional (2D) material derived from graphene by introducing functional groups with covalent C–O bonds,^19^ either in or out of the sp2-bonded carbon atoms arranged in a hexagonal lattice. GO has presented itself as an exciting material^20−22^ for biological and biomedical applications,^21,23−26^ including bioimaging, biosensing, and drug delivery. GO has a large surface area characterized by the presence of many functional groups such as hydroxyl, carboxyl, and carbonyl groups.^27,28^ These functional groups can be further modified to achieve enhanced biocompatibility, aqueous solubility, and high cargo loading capacity.^27,29−32^ The large surface area to volume ratio of GO reduces cytotoxicity and supports effective endocytosis due to its increased interactions with cells.^33^ GO and its derivatives, obtained by covalent modification of surface functional groups with polymers such as polyethylene glycol (PEG), chitosan, dextran, and dendrimers (e.g., polyamidoamine, PAMAM), have been used to achieve gene silencing in various cancer cells.^29,34−39^ We have previously shown that GO-mediated delivery of siRNA targeting CD47 in different cancer cell lines downregulated CD47 protein expression, which can inhibit the antiphagocytosis “do not eat me” signal. This increases their susceptibility to phagocytic clearance by macrophages.^35,36,40^ Additionally, when CD47 was knocked down, there was a significant increase in the production of IL-6, TNF-α, IL-1β, and IL-8 in the culture media compared to the control (IL-8, IL-6, and TNF-α showed a remarkable increase of over 65 times, and IL-1β increased >40 times, compared to the control: cancer cells in coculture without transfection), strongly implying that their main function is to target and eliminate cancer cells.^35^ Furthermore, GO size, surface modifications (PEG and PAMAM), and cargo-to-GO ratios play important roles in transfection efficiency of CD47_siRNA in both two-dimensional (2D) and three-dimensional (3D) cultures of lung cancer cell lines.

In this research, our goal is to establish in vitro models of chronic inflammation using murine small intestines (enteroids) and large intestines (colonoids). To induce inflammation in these 3D organoid models, we employ well-known inflammatory agents, including IL-1β, TNF-α, and lipopolysaccharide (LPS).^41,42^ We chose 3D organoid models for their ability to closely mimic the complex cellular interactions found in vivo.^43^ Using graphene oxide (GO) as a nanocarrier with various sizes and surface modifications, we specifically deliver TNF-α_siRNA to the organoids. This approach targets and silences the TNF-α gene within the organoids, potentially offering a path to address IBD.

## Materials and Methods

### Chemicals and Reagents

Mouse colonoids were kindly donated by the group of Simon Hirota at the University of Calgary. Mouse intestinal organoids, IntestiCult Organoid Growth Medium (mouse), and gentle cell dissociation reagent (GCDR) were purchased from Stem Cell Technologies.

Phosphate-buffered saline (PBS) 1× solution (pH 7.4), Dulbecco’s Modified Eagle Medium/Nutrient Mixture F-12 (DMEM/F12), Opti-MEM transfection media, Silencer Select Negative Control No. 1 siRNA (cat# 4390843), Silencer Select TNF-α_siRNA (cat# 4390771), lipofectamine RNAiMAX Transfection Reagent, Click-iT TUNEL Alexa Fluor 647 kit, Scigen Tissue-Plus O.C.T. Compound, 4% paraformaldehyde (PFA) in PBS, Falcon 96-well Clear Flat Bottom TC-treated Culture Microplate, Falcon 24-well Clear Flat Bottom TC-treated Multiwell Cell Culture Plates, recombinant anti-ChgA antibody, recombinant anti-lysozyme antibody, recombinant anti-Lgr5 antibody, Goat antirabbit Alexa Fluor 488, Goat antimouse Alexa Fluor 555, Hoechst 33342, IL-1β recombinant protein, TNF-α recombinant protein, Power SYBR Green RNA-to-C_T_ 1-Step Kit, Mouse ELISA kits of TNF-α, IL-1β, and IL-6, as well as 10% normal goat serum were purchased from Thermo Fisher Scientific. Matrigel Matrix Growth Factor Reduced (Phenol Red-Free, LDEV-Free) for organoid culture was purchased from Corning. Bovine serum albumin (BSA) and lipopolysaccharides (LPS) from *Escherichia coli* (O127:B8) were purchased from Sigma-Aldrich. Cell counting kit 8 (WST-8), recombinant anti-E-cadherin antibody, recombinant anti-villin antibody, recombinant anti-TLR4, recombinant anti-TNFR1, and recombinant anti-MUC2 antibody were purchased from Abcam. RNeasy mini kit and Quantitect Primer assays for Hprt1 and Tnf-α were purchased from Qiagen.

Single-layered GO was synthesized using the modified Hummer’s method and filtered to produce big GO (bGO, >650 nm).^27^ Then, sonication was performed (probe sonicator, Cole Parmer) at 10 W for nine intervals of 10 min on/off in a 0 °C water bath to create small GO (sGO, mainly between 50 and 80 nm). bGO and sGO were chemically modified by the addition of six-arm amine-terminated polyethylene glycol (PEG, 25 mg, Jenkem Technology, 15 kDa) and PAMAM dendrimers (10 μL, 4.0 generation, primary amine surface area, 10.1 w/w% in water, 10 kDa, Dendritech Inc.) as previously reported.^36^ Modified GO dispersions of large size (bmGO > 650 nm; 0.7 mg/mL) and small size (smGO ∼ 100 nm; 1 mg/mL) were obtained and used as a stock for further characterization and transfection experiments. Characterizations of GO (such as atomic force microscopy (AFM) imaging, Raman spectroscopy, and X-ray photoelectron spectroscopy), with toxicity assessment, were carried out and reported previously.^44,45^ Further details of the preparation of GO nanocarriers can be found in the Supporting Information of previously published references.^36^

### Characterization of GO Carriers

Briefly, atomic force microscopy topography imaging was carried out using the MultiMode AFM with a NanoScope V controller (Bruker Nano Surfaces Division, Santa Barbara, CA, USA), in the Peak Force QNM mode. The peak force with which the tip taps the sample surface was always kept at the lowest stable imaging level of 200–400 pN. Silicon nitride ScanAsyst-Air AFM probes (Bruker AFM Probes, Camarillo, CA, USA) were used in peak force feedback measurements. Their manufacturer specified that typical tip diameter and spring constants are 2 nm and 0.4 N/m, respectively. While images of sizes of up to 20 μm × 20 μm were acquired to ensure good homogeneity without overlapping of flakes of GO and modified GO, all the images used to measure the size of the flakes were 0.5–2 and 2.5–5 μm in scan size, for small-GO (or GO-conjugates) and big-GO (or GO-conjugates), respectively, acquired with 512 × 512 pixels or 1024 × 1024 resolution. The AFM probe cantilever was vertically oscillated at 2 kHz at a lateral scan rate of 0.8–1.4 Hz.

The chemical properties modified GO were carried out by using a Fourier transform infrared (FTIR) spectrometer (Nicolet 6700, ThermoFisher) equipped with a substrate sample holder accessory (that allows measurements of sGO, smGO, and bmGO thin films on silicon substrates), in the range of 400 to 4000 cm^–1^ with a resolution of 4 cm^–1^. The generation of amide groups, increased intensity of C–O, and decreased intensity of C=O groups in GO, confirmed the successful formation of GO–PEG-PAMAM.

Previously, dynamic light scattering (DLS) measurements were conducted to determine the size distribution and charges of smGO, sGO, and bmGO. The confirmation of successful loading of siRNA was measured by the changes of surface charges from positive potential values to around −18 to −20 mV by the DLS. Detailed results and figures can be found in our previous publication (Figures S1, S2, and S5 in ref (36)).

### Culture of Enteroids and Colonoids

Cryopreserved mouse enteroids and colonoids were thawed for 2.5 min in a 37 °C water bath and resuspended in DMEM/F12 with 1% BSA. The organoids were then centrifuged at 200 × *g* for 5 min and washed with cold DMEM/F12 with 1% BSA. After the wash, the organoids were resuspended in cold Matrigel and placed on ice. Twenty-five microliters of Matrigel containing approximately 200 organoid fragments was placed at the center of a well of 24-well plate, which has been prewarmed at 37 °C in a tissue culture incubator. Once the Matrigel was set (approximately 5 min), the plate was incubated for 30 min upside down at 37 °C and 5% CO_2_ in a tissue culture incubator to allow for dome formation. Finally, 500 μL of IntestiCult organoid media was added. The organoids were cultured for 10 days, with media changes performed every 48 h. For induction of inflammation to mimic IBD, 3 d old organoids were treated with 100 ng/mL LPS, 10 ng/mL IL-1β, and 10 ng/mL TNF-α for 7 d.^41,42^ A full media (supplemented with a cytokine cocktail) change was performed every 48 h.

### Monitoring Growth of Organoids

Brightfield microscopy was performed to follow the progression of intestinal organoid (enteroid and colonoids) growth. The morphology of the organoids was characterized by using immunohistochemistry. Frozen sections (10 μm) were prepared and blocked with 10% goat serum. The sections were then incubated with anti-ChgA, anti-Muc2, anti-Lgr5, anti-E-cadherin, anti-villin, anti-lysozyme, anti-TLR4, and anti-TNFR1 antibodies overnight at 4 °C. Fluorescence labeling was performed with secondary antibodies conjugated with Alexa Fluor 488 and 555. Hoechst was used for labeling the nuclei. The organoids were analyzed using an Olympus confocal laser scanning microscope, with a 10× objective.

### Passage of Organoids

The organoids were passaged on day 10. Briefly, the cell culture media was removed, and the Matrigel dome was incubated with 1 mL of GCDR for 1 min at RT. The GCDR was then pipetted up and down using a 1 mL pipet tip to break down the Matrigel dome, which was then transferred to a 15 mL tube and incubated at RT for 10 min on an orbital shaker. The tube was then centrifuged to remove the GCDR, and the organoid fragments were resuspended in 2 mL of DMEM/F12 with 1% BSA. A 10 μL droplet was taken to calculate the number of organoids, and approximately 200 organoids were suspended in 25 μL. Matrigel was added to each well of a 24-well plate as described earlier to culture.

### Cytotoxicity of GO on Enteroids and Colonoids

Organoids were passaged and 50 organoids resuspended in 10 μL of Matrigel were added carefully to the center of each well of a 96-well plate. The organoids were cultured with 100 μL of media for 24 h at 37 °C and 5% CO_2_. Then, the organoids were treated with different concentrations of sGO, smGO and bmGO (0.25, 1, 4, and 16 μg/mL). Organoids with no treatments were used as controls. After 48 h, 10 μL of WST-8 was added to each well, and then, the plates were incubated in a tissue culture incubator for 4 h. The absorbance was measured at 450 nm by using a microplate reader. Cell viability was calculated using the following formula:
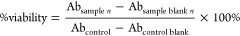
where Ab_sample_ is the absorbance of the organoids with *n* μg/mL GO treatments, Ab_sample blank *n*_ is the absorbance of the same *n* μg/mL GO without any organoids, Ab_control_ is the absorbance of organoids with no treatments, and Ab_control blank_ is the absorbance of media with Matrigel but no organoids.

### Transfection with TNF-α_siRNA

To prepare TNF-α_siRNA with the different formulations of GO (TNF-α_siRNA/GO), transfection mixes were prepared by mixing TNF-α_siRNA in 250 μL of Opti-MEM transfection media with each of the GO formulations (i.e., sGO, smGO, and bmGO) (1:1 and 3:1 siRNA:GO) and incubating for 1 h at RT. IBD organoids and control organoids were cultured in 24-well plates with organoid culture media. After 7 days of culture, the media was removed, and fresh Opti-MEM media (without proinflammatory cocktail) was added. Next, different transfection mixtures with a concentration of 0.25 μg/mL were added to the wells, and the plates were incubated in a tissue culture incubator for 48 h. A universal negative control (nontargeting control or NTC) was used to determine whether changes in gene expression are nonspecific. Transfection of TNF-α_siRNA with lipofectamine RNAiMAX was performed in accordance with the manufacturer’s instructions. Subsequent treatments with sGO and smGO were carried out as previously described without the addition of TNF-α_siRNA.

### Reverse Transcription Quantitative Polymerase Chain Reaction (RT-qPCR)

Transfection efficiency was assessed via the reverse transcription quantitative polymerase chain reaction (RT-qPCR) for TNF-α expression. Organoids from three wells of each treatment were harvested and pooled using GCDR. RNA was isolated using RNeasy Mini Kit and RT-qPCR was carried out with Power SYBR Green RNA-to-CT 1-Step Kit. The expression of TNF-α target gene was normalized to reference gene Hprt1 to obtain ΔCq value, which was then exponentially transformed to ΔCq expression value (ΔCq expression = 2^–ΔCq^). The mean ΔCq expression was then normalized to NTC to find the ΔΔCq. Finally, % knockdown was calculated by subtracting the ΔΔCq from 1 (defined by level of expression for the untreated sample) and multiplying by 100.

### Cytokine Concentration Measurements

Cell culture media from untreated, IBD, and siRNA-transfected organoids, as well as those treated with sGO and smGO, were spun down at 2000 × *g* to remove debris. The supernatant was collected and then frozen at −80 °C for storage. Frozen media were later thawed and used to perform ELISA with mouse IL-1β, IL-6, and TNF-α kits as per the manufacturer’s instructions.

### Cell Apoptosis Measurements

Frozen sections (10 μm) obtained from prepared organoids were stained for TUNEL using a Clict-iT TUNEL Alexa Fluor 647 Imaging Assay kit, following the manufacturer’s instructions. The imaging was conducted using a confocal laser scanning microscope. The quantification of apoptotic cells within each organoid was performed by counting the cells positive for both TUNEL and Hoechst staining. TUNEL stain outside the Hoechst region was excluded from the count, as these signals were likely background noise captured by residual Matrigel from the organoid culture.

### Morphological Scoring

Untreated, proinflammatory, and siRNA-treated organoids were visualized using an ECHO Revolve bright-field microscope, with 10× objective. Organoids showing an irregular epithelial layer with blebbing were counted. The morphological score was calculated by the ratio of impaired organoids observed to the total number of organoids within the field of view.

### Statistical Analysis

All the results described here are from at least three independent experiments. Data were analyzed using GraphPad Prism 8 software. Unpaired two-tailed *t* tests were done for single comparisons between two groups. A one-way analysis of variance (ANOVA) was conducted for more than two groups, followed by a post hoc test and adjustment for multiple comparisons. A value of *p* < 0.05 was set as the limit of statistical significance.

## Results and Discussion

### Organotypic Morphology of Mouse Enteroids and Colonoids

In recent years, organoids have gained popularity for testing drugs and for modeling diseases.^46^ These organoids, which are defined as in vitro 3D cell clusters derived from adult or pluripotent stem cells capable of self-renewal and self-organization while retaining morphology and functional similarities to the tissue of origin, have emerged as invaluable tools in biomedical research.^46^ Specifically, intestinal organoids, typically derived from intestinal crypts containing intestinal stem cells, offer a unique advantage. They not only self-renew and give rise to various cell types found in vivo but also can be maintained in culture over extended periods. This long-term culture capability allows us to mimic the complex interactions among different cell types during both homeostasis and disease, providing a platform that is more physiologically relevant.^46,47^

**Figure 1:**
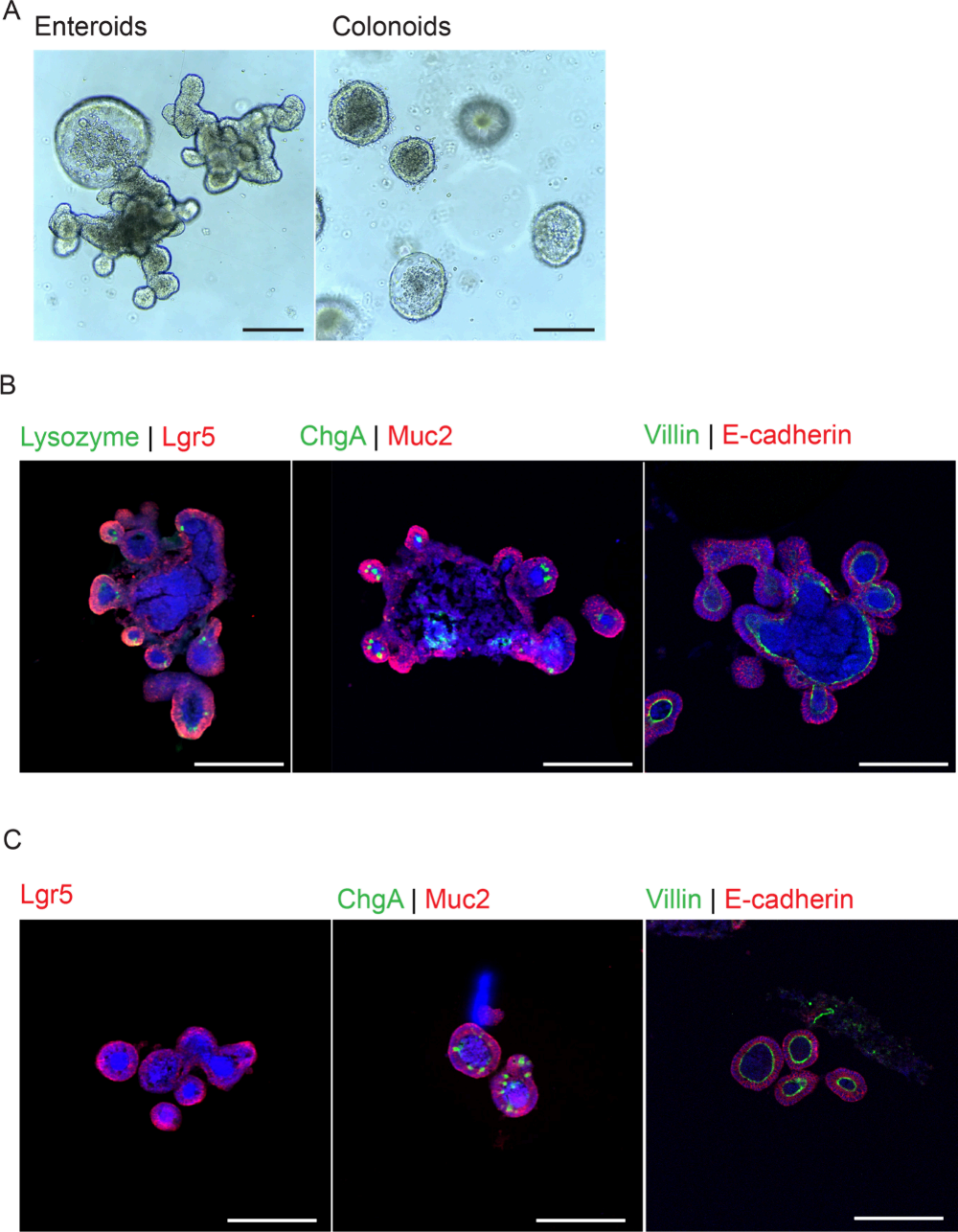
Intestinal organoids show organ specific cell types and morphology. (A) Bright-field images of enteroids and colonoids at day 5 of culture. Scale bars measure 500 μm. Immunofluorescence images of day 10 (B) enteroids and (C) colonoids. Lgr5 (red), E-cadherin (red), villin (green), ChgA (green), and Muc2 (red) show the presence of epithelial stem cells, enterocytes, goblet cells, and enteroendocrine cells. Paneth cells are shown with lysozyme (green) in panel (B) and nucleus (Hoechst, blue) in panels (B) and (C). Scale bars measure 500 μm.

In our study, we harnessed the power of these intestinal organoids to investigate their organotypic morphology and functional characteristics. By day 5 of the culture, enteroids displayed budding structures reminiscent of in vivo tissue, while colonoids formed circular spheroids. Both organoids exhibited a well-defined epithelial basal layer and lumen (as shown in Figure 1A). The intestinal epithelium, known for its single layer of rapidly regenerating epithelial cells, plays a pivotal role in maintaining gut health.^48^ This turnover process is facilitated by a group of stem cells, notably identified by their expression of the Wnt-targeted surface protein, Lgr5. In the small intestine, Paneth cells located adjacent to these stem cells produce Wnt molecules that sustain the stem cell phenotype. The Lgr5^+ve^ stem cells proliferate and generate a large number of transit amplifying cells (TACs), which, in turn, differentiate into different gut cell types, including enterocytes, enteroendocrine cells, and mucin-producing goblet cells.^49^ However, the large intestines lack Paneth cells, and instead, the Lgr5^+ve^ stem cells in the colon receive signals from Wnt molecules that originate from the underlying mesenchyme, thereby maintaining the stem cell homeostasis.^50^

Our organoid culture system aimed to replicate this intricate cellular structure and communication in an in vitro setting. To assess organotypic morphology, we conducted staining for key markers such as Lgr5, lysozyme, Muc2, ChgA, villin, and E-cadherin^51^ (as illustrated in Figure 1B–C). Both enteroids and colonoids exhibited enterocytes expressing E-cadherin at the basolateral membrane and villin at the apical surface, akin to in vivo conditions. Additionally, enteroids were found to display lysozyme expression, indicating the presence of Paneth cells, a feature consistent with the in vivo observations. Moreover, both organoids contained enteroendocrine cells and goblet cells, as demonstrated by the presence of ChgA and Muc2 (Figure 1B,C). Furthermore, we conducted an in-depth characterization of the organoids to assess the expression of key receptors, namely, TNF-α receptor tumor necrosis factor receptor 1(TNFR1) and toll-like receptor 4 (TLR4). TNF-α mediated inflammation is propagated through TNFR1, expressed by intestinal epithelial cells.^52−55^ TLR4, a pattern recognition receptor, identifies conserved pathogen-associated molecular patterns such as LPS^56,57^ and is implicated in ulceration and tissue destruction in IBD.^58^ Our findings revealed robust expression of TNFR1 in both enteroids and colonoids, emphasizing its significance. However, TLR4 expression was limited to a small subset of cells, aligning with previous reports highlighting the generally low expression levels of TLR4 in intestinal epithelial cells (see Figure S1).^58^

Overall, this shows that both enteroids and colonoids had not only morphological but also functional similarities to their in vivo counterparts, providing a robust platform for our subsequent investigations into the therapeutic potential of graphene oxide-mediated TNF-α_siRNA delivery in the context of chronic inflammation, particularly IBD.

### Characterization of GO Formulations

**Figure 2:**
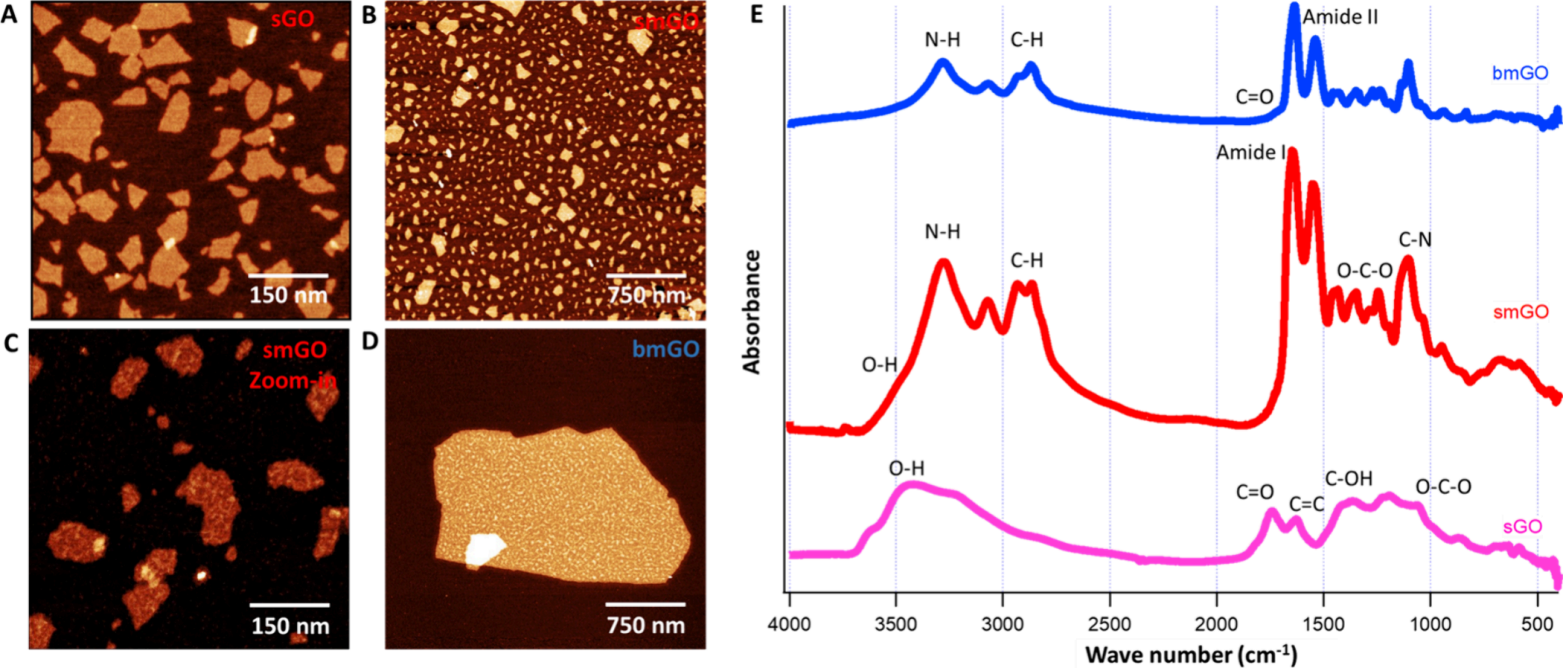
Chemical modification of graphene oxide. AFM topography images of (A) sGO, (B) smGO, (C) zoomed-in smGO, and (D) bmGO demonstrate single-layer GO flakes. (E) FT-IR spectra of sGO, smGO, and bmGO show functional groups such as hydroxyl (O–H), carbonyl (C=O), and carbon–carbon double bonds (C=C) on GO reacting with PEG and PAMAM and with decreased absorbance intensity on smGO and bmGO. New functional groups such as amide groups and sharp C–O were evidently observed on smGO and bmGO.

The modification of GO involved the incorporation of branched amine-functionalized PEG and dendrimers (PAMAM) to optimize binding sites for siRNA. This modification induced morphological changes, including increased roughness on GO flakes in smGO and bmGO upon conjugation, as evidenced in Figure 2A–D, contrasting with their unmodified GO counterparts. This modification process has been corroborated by FT-IR spectroscopy (Figure 2E). The emergence of amide groups (amide I at 1645 cm^–1^, amide II at 1555 cm^–1^), N–H at 3400 cm^–1^, and decreased intensity of carbonyl (C=O, 1720 cm^–1^), hydroxyl (O–H, 3200–3500 cm^–1^), and C–OH (1415 cm^–1^) groups in GO were observed. These suggested that the epoxide and ketone groups in GO may undergo nucleophilic reaction with the amine groups of PEG and PAMAM to form covalent bonds, confirming the successful formation of GO–PEG-PAMAM (i.e., smGO and bmGO). These chemically engineered GO formulations have been reported to amplify transfection efficiency.^59^ This enhancement is attributed to their inherent positively charged nature due to primary amine functional groups, which are similar to the conditions prevalent in physiological culture media. Consequently, this characteristic bestows heightened stability and augmented payload loading capacity, especially advantageous for molecules like siRNA.^60^ Noteworthy are the pivotal electrostatic interactions between PAMAM and siRNA, which is instrumental in elevating siRNA knockdown efficiency.^61^

### Dose-Dependent Cytotoxicity of GO Formulations on Organoids

**Figure 3:**
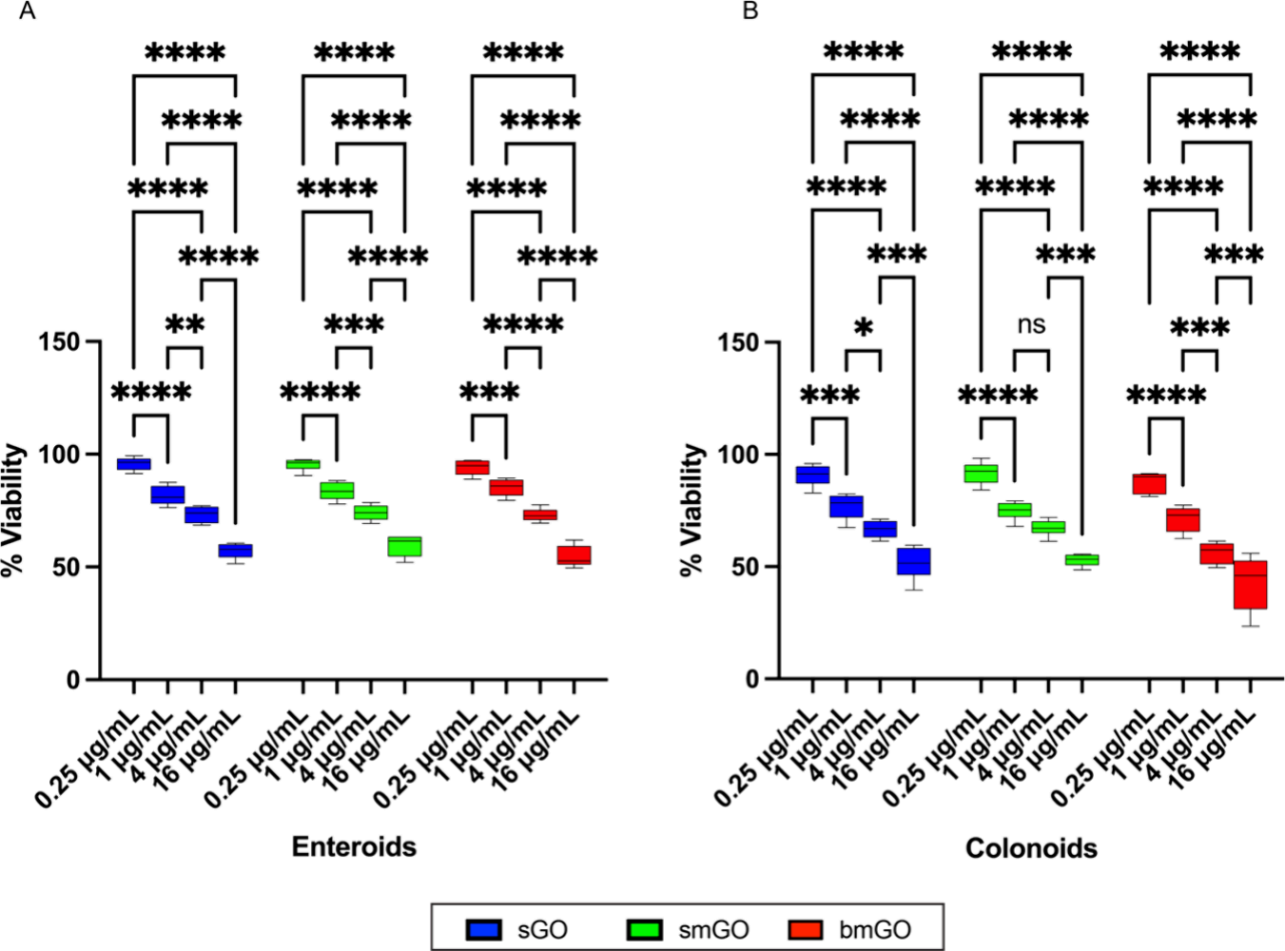
Increasing concentrations (0.25, 1, 4, and 16 μg/mL) of GO formulations impair viability. Relative viability of (A) enteroids and (B) colonoids determined using WST-8 assay. Values on each graph are shown as mean ± SD of three independent experiments each with replicates (*n* = 3). Statistical significance was determined with one-way ANOVA. *P* < 0.05 was considered significant. *P < 0.05; **P < 0.01; ***P < 0.001; ****P < 0.0001. *P* > 0.05, ns: not significant.

Organoids were treated with four different concentrations (0.25, 1, 4, and 16 μg/mL) of sGO, smGO, and bmGO to evaluate the viability and determine the nonlethal concentration for transfections. Exposure time of 48 h was chosen as transfections with commercially available kits such as lipofectamine typically require 48 h, and the concentrations were selected based on our previous work. We and others^62,63^ have previously reported that the cytotoxicity of GO formulations can vary based on cell lines. For example, 3D spheroids generated from lung (A549) and leukemia (NB4) cancer cell lines showed noticeable toxicity only at 16 and 64 μg/mL but not at 1 and 4 μg/mL. However, spheroids generated from fibroblast cell line (NIH 3T3) showed cell viability less than 80% at a GO treatment concentration of 4 μg/mL.^62^ In contrast to previous reports, both enteroids and colonoids showed a loss of viability in a dose-dependent manner (Figure 3A,B). This is likely due to clonal nature of intestinal organoid culture, which promotes cellular homogeneity and increased sensitivity.^64^ The lowest concentration of 0.25 μg/mL did not negatively impact cell viability for any of the GO formulations, showing viability levels of >94% for enteroids and 87% for colonoids for all formulations. Increasing concentrations of all three GO formulations lead to reduced viability levels. Particularly, the higher concentrations of 4 and 16 μg/mL showed cell viability levels below 75% for both enteroids and colonoids. Thus, subsequent experiments were conducted with 0.25 μg/mL GO formulations.

### Proinflammatory Effectors Induced Inflammatory Phenotype

**Figure 4:**
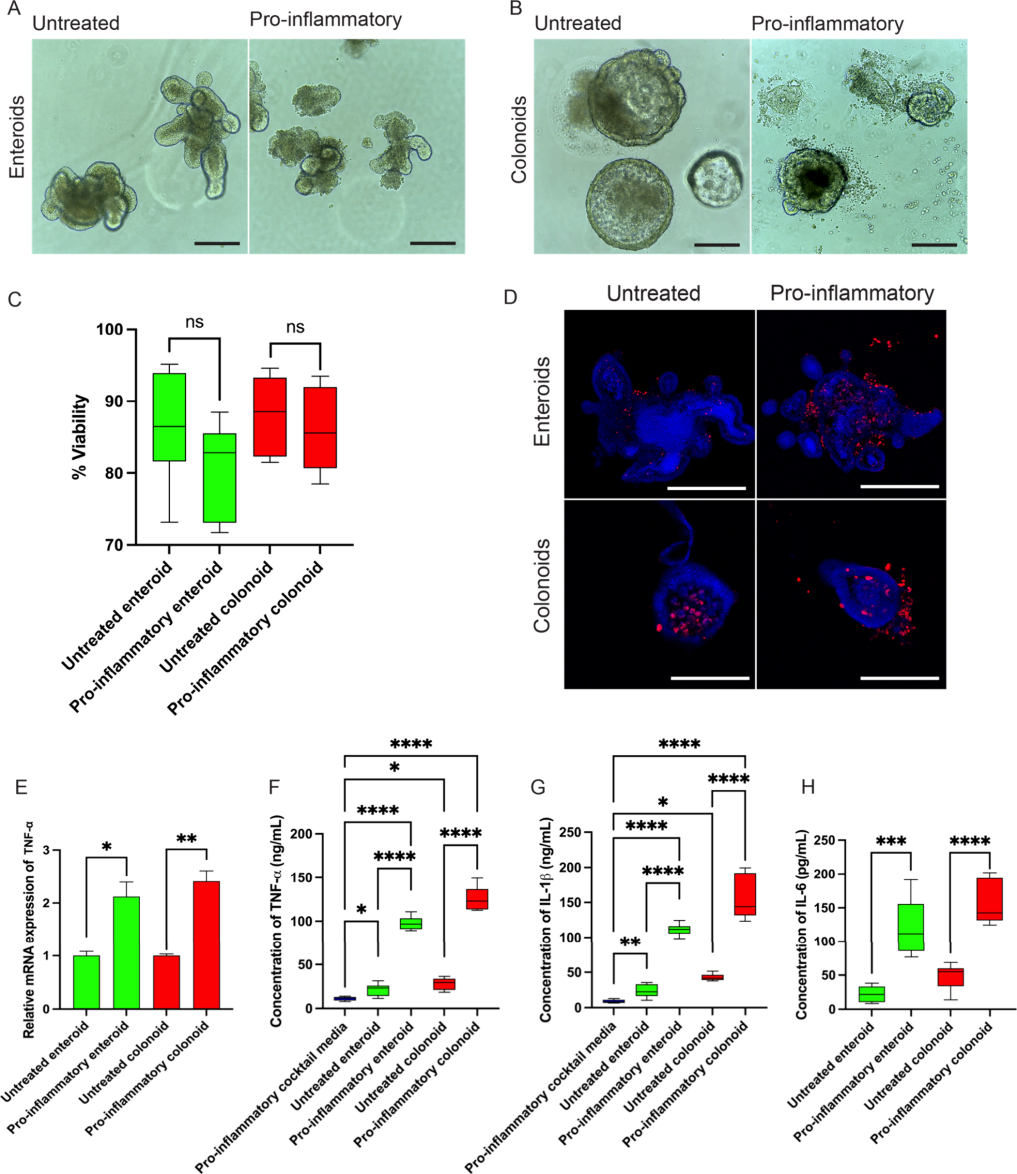
Treatment with proinflammatory factors gave rise to proinflammatory phenotypes. Brightfield images of (A) enteroids and (B) colonoids after 6 days of induction. Scale bars measure 500 μm. (C) Relative viability of intestinal organoids was determined using the WST-8 assay. (D) Immunofluorescence images of intestinal organoids showing TUNEL^+ve^ apoptotic cells (Hoechst, blue and TUNEL, red). Top panel: enteroids. Scale bars measure 250 μm. Bottom panel: colonoids. Scale bars measure 100 μm. (E) Relative fold change of mRNA expression for TNF-α after 7 days of induction. Concentrations of (F) TNF-α, (G) IL-1β, and (H) IL-6. Values on each graph are shown as mean ± SD of six independent experiments (*n* = 6). Statistical significance was determined with *t* test (for comparing two groups) and one-way ANOVA (for comparing three groups or more). *P* > 0.05 was considered not significant (ns). *P < 0.05; **P < 0.01; ***P < 0.001; ****P < 0.0001.

Intestine is a highly intricate organ system that comes into contact with a multitude of foreign substances ingested in the diet. The intestinal epithelia serve as a crucial barrier, effectively separating the internal organs from luminal foreign particles and bacteria. IBD is characterized by the long-term inflammation of this intestinal epithelia.^5−7^ To induce an inflammatory phenotype in our organoids, we exposed them to a specific inflammatory cocktail consisting of LPS, IL-1β, and TNF-α for 7 days.^41,42^ The selected factors and their concentrations were chosen based on previously reported examples regarding the regulation of cytokines and other associated inflammatory factors. For example, different combinations of small molecules such as azoxymethane, bacterial components (i.e., LPS and flagellin), and cytokines have been used to generate IBD organoid models.^41,42^ In IBD, there is an overproduction of IL-1β and TNF-α that contributes to chronic inflammation. Both cytokines were initially included to mimic the inflammatory milieu characteristic of IBD. However, it failed to produce the characteristic morphological blebbing,^41^ which was rectified by the addition of LPS.^41,42^ This treatment had noticeable effects on the organoids’ morphology and health. Compared to control organoids, those exposed to the inflammatory cocktail exhibited reduced size and significant characteristic structural irregularities, including pronounced blebbing and a disrupted epithelial layer (as shown in Figure 4A,B).^41,42^ Organoids treated with proinflammatory factors had three times more morphologically impaired organoids compared to the untreated controls (Figure S2A). These alterations closely resembled changes observed in colon organoids derived from IBD patients, highlighting the effectiveness of our induced inflammation model.^65,66^

Further analysis revealed a trend toward reduced cell viability in the proinflammatory organoids compared to the control group (from approximately 90 to 80%, *p* > 0.05, as indicated in Figure 4C). This is likely because the in vitro induction of IBD requires further refinements in terms of inflammatory factor concentrations or exposure duration. D’Aldebert et al. observed that while colonoids derived from IBD patients showed reduced viability as measured by metabolic activity for 9 days, healthy organoids that were treated with inflammatory factors showed a reduction in viability only immediately after the stimulation. This effect did not persist for more than 72 h.^41^ To visualize the cells undergoing apoptosis, we conducted TUNEL staining. While both the treated and control organoids exhibited TUNEL-positive (TUNEL^+ve^) cells in the lumen region (which is similar to in situ),^51,67,68^ the treated group displayed more TUNEL^+ve^ cells within the epithelium (as depicted in Figure 4D), signifying disturbances in normal tissue homeostasis. Quantification of the TUNEL^+ve^ cells revealed an increase in the number of apoptotic cells resulting from the induction of inflammation (see Figure S2B), similar to what has been shown for colonoid models previously.^41^

Moreover, incubation with the proinflammatory cocktail resulted in a notable upregulation of TNF-α mRNA by 2.1-fold in enteroids and 2.4-fold in colonoids (as shown in Figure 4E). Consistent with previous studies on acute inflammatory injury induced by high LPS levels, similar upregulation of TNF-α expression (up to 2.5-fold) was observed after 24 h of exposure (as shown in Figure 4E).^69^ In the progression of IBD, there is an abnormal overproduction of various inflammatory cytokines by epithelial cells and innate immune cells.

To further validate this induction model, we measured the levels of secreted proteins, including TNF-α (in ng/mL), IL-1β (in ng/mL), and IL-6 (in pg/mL), in the cell culture supernatant, as shown in Figure 4F–H. Given that the proinflammatory cocktail contained both TNF-α and IL-1β, a comparison of secreted cytokine levels was made among treated organoids, untreated controls, and the proinflammatory media without any organoids (as shown in Figure 4F,G). The results demonstrated that untreated controls maintained a basal level of cytokine expression. Importantly, the inflammatory cocktail induced increased secretion levels of TNF-α, IL-6, and IL-1β compared with the untreated controls. This observation aligns with previous research indicating that treatment of organoids with 64 ng/mL of TNF-α induced the production of TNF-α and associated inflammatory cytokines.^70^ In summary, these findings collectively establish the successful induction of a robust inflammatory phenotype closely resembling that observed in IBD. This model serves as a valuable tool for investigating the mechanisms underlying chronic inflammation and evaluating potential therapeutic interventions.^7,41,71^

### smGO-Mediated siRNA Delivery

**Figure 5:**
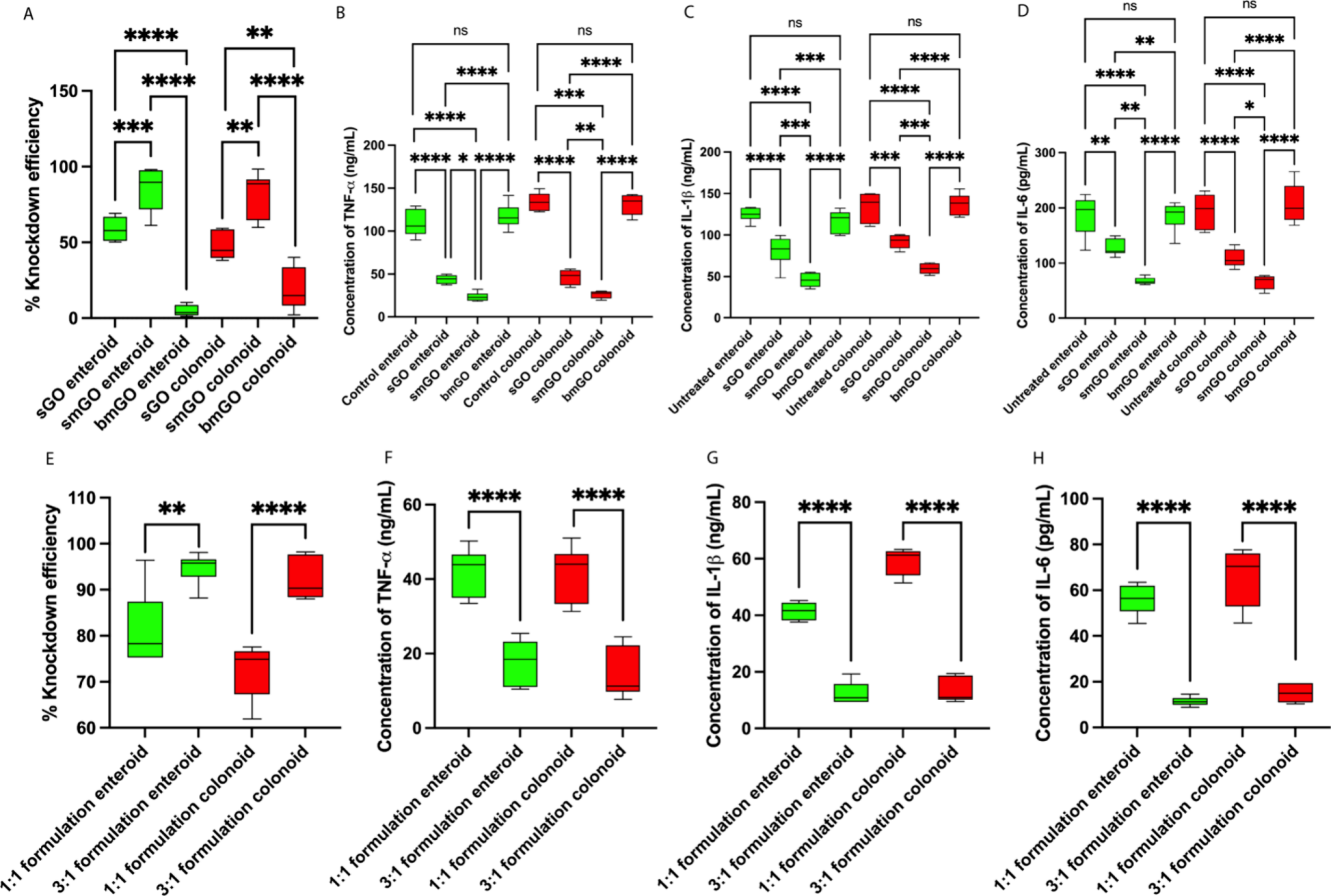
The highest degree of transfection was demonstrated by smGO. (A) Knockdown efficiency for TNF-α after 48 h of transfections. Increased knockdown efficiency translated to reduced cytokine secretion of (B) TNF-α, (C) IL-1β, and (D) IL-6. (E) Knockdown efficiency for TNF-α after transfections of enteroids and colonoids with 3:1 siRNA:smGO. Increased knockdown efficiency with the 3:1 formulation resulted in further reduction of cytokine secretion for (F) TNF-α, (G) IL-1β, and (H) IL-6. Values on each graph are shown as mean ± SD of six independent experiments (*n* = 6). Statistical significance was determined with *t* test (for comparing two groups) and one-way ANOVA (for comparing three groups or more). *P* > 0.05 was considered ns: not significant. *P < 0.05; **P < 0.01; ***P < 0.001; ****P < 0.0001.

After successfully inducing a proinflammatory phenotype in the intestinal organoids, we proceeded to evaluate the gene silencing of TNF-α with a GO-mediated TNF-α_siRNA transfection approach. The induced organoids were subjected to transfection with TNF-α_siRNA, using three different GO formulations: sGO, smGO, and bmGO (all at a 1:1 siRNA:GO ratio). Following 48 h of transfection, transfection efficiency was calculated based on gene expression of TNF-α. The highest knockdown efficiency was attained in organoids transfected with smGO (85% for enteroids and 82% for colonoids) compared to that of sGO (59% for enteroids and 48% for colonoids) and bmGO (5% for enteroids and 19% for colonoids), as shown in Figure 5A.

It is worth noting that in our previous work, bmGO-mediated transfection of CD47_siRNA demonstrated over 40% transfection efficiency in 2D cell culture models using NB4 and HL-60 leukemia cell lines (compared to the controls).^36^ Here, the absence of bmGO-mediated transfection in 3D intestinal organoids can be attributed to the complex culture modality associated with their propagation. Unlike conventional monolayer cultures, 3D intestinal organoids are embedded within Matrigel droplets, which can act as a barrier to nanoparticle diffusion and uptake. Additionally, the basal cells of organoids form a basement membrane that can further impede nanoparticle penetration.^72,73^

In the context of chronic or acute inflammation due to injury or infection, cells in the affected tissue produce elevated and high levels of TNF-α cytokine, triggering the production of other inflammatory cytokines, thereby intensifying the inflammatory response.^5,74,75^ Inhibition of TNF-α has been shown to restore immunosuppressive capabilities, reduce the release of other cytokines, such as IL-1β and IL-6, and attenuate inflammatory damage in IBD.^14,76,77^ Thus, we further validated gene silencing by evaluating secreted cytokine levels, revealing significant downregulations of TNF-α and its downstream effectors, IL-1β and IL-6 (as shown in Figure 5B-D), with smGO exhibiting the most pronounced reduction, while bmGO-mediated transfection had no effects compared to the controls. Similar downregulation of TNF-α (4-fold) and IL-6 (3-fold) cytokines has been reported for LPS-induced mouse macrophages upon transfection with TNF-α_siRNA.^14^ Oral delivery of TNF-α_siRNA using type B gelatin nanoparticles in dextran sodium sulfate induced mouse model of colitis has shown approximately 50% reduction of IL-6 serum protein levels.^76^ Our results are also in agreement with these in vivo models of IBD, where gene silencing of TNF-α has been shown to downregulate both the gene and protein expressions of TNF-α, IL-1β and IL-6.^76,78^ Morphological scoring revealed a lower occurrence of organoids with morphological defects in those treated with TNF-α_siRNA_smGO compared to that of the untreated inflamed organoids (see Figure S3A). In addition, the TNF-α_siRNA_smGO also showed a reduction in the number of apoptotic cells compared to the respective controls (Figure S3B,C), suggesting a positive therapeutic outcome following transfection.^62^

To discount any anti-inflammatory effects of sGO and smGO, inflamed organoids were treated with sGO and smGO (without any siRNA) and the expression of TNF-α was evaluated. The mRNA expression of TNF-α in organoids treated with sGO and smGO showed no significant difference compared to untreated organoids (Figure S3D). Similarly, there were no observable changes in the levels of secreted proteins of TNF-α, IL-1β, and IL-6, between the treated and untreated groups (Figure S3E–G). Taken together, these findings indicate that GO formulations do not possess inherent immunomodulatory effects, and the observed anti-inflammatory effects of sGO and smGO-mediated siRNA delivery are primarily due to the efficient transfection of TNF-α_siRNA.

To establish a benchmark of transfection efficiency, we also transfected the inflamed organoids with the commercially available and commonly used lipofectamine RNAiMAX reagent. The knockdown efficiency with smGO, reaching 85% for enteroids and 81% for colonoids in this specific test, surpassed that of lipofectamine-mediated transfection (which achieved 69% for enteroids and 63% for colonoids; see Figure S3H). This outcome suggests an improved utility of smGO-mediated delivery in comparison to lipofectamine.

Based on our previous findings that increasing the siRNA to nanocarrier ratio enhances transfection efficiency,^62^ we conducted TNF-α_siRNA transfection using a formulation containing a 3:1 siRNA:smGO ratio. This choice was also informed by the superior transfection efficiency observed with smGO in the 1:1 siRNA:GO formulations (Figure 5A). The 3:1 formulation resulted in a higher knockdown efficiency (94% for enteroids and 92% for colonoids) compared to the 1:1 formulation (81% for enteroids and 72% for colonoids) (as depicted in Figure 5D). Similarly, reduced levels of secreted cytokines were observed in the cell culture supernatant (Figure 5F–H).

As a 2D material, the GO boasts an exceptionally high surface area, facilitating the efficient loading of drugs, ligands, nucleic acids, peptides, and proteins.^79^ The large surface area of GO flakes makes it amenable to modifications with various functional groups, enabling targeted delivery through different modes of administration such as oral and injectable rounts.^79^ GOs that are functionalized with chitosan, poly(acrylic acid) (PAA), and thiols have demonstrated the capacity to improve bioavailability and mucoadhesive properties of GO nanoparticles.^79−81^ In particular, thiolated GOs exhibited improved mucoadhesive properties and prolonged residence time in mucosal tissues, such as the gastrointestinal tract, offering the potential for targeted delivery of drugs and nucleotides to specific areas affected by IBD.^80^ PAA-modified GOs were shown to be pH sensitive and have proven valuable for intracellular protein delivery via oral administration in intestinal tissue.^82^ These GOs, grafted with pH-sensitive PAA, effectively prevented the premature release of cargo such as bovine serum albumin (BSA) in the highly acidic pH of the stomach. Simultaneously, they facilitated the release of cargo in the desired basic pH of intestines enabling site specific delivery.^82^ Furthermore, GOs have also demonstrated efficacy as nanocarriers when administered via injectable routes. Polyethylene glycol decorated GOs loaded with chemotherapies, such as cisplatin and doxorubicin, displayed efficient targeting of cancer cells and reduced off-target effects on healthy cells when administered intravenously.^83^ The attachment of biorecognition ligands like antibodies, peptides, and specific DNA sequences imparts active targeting while minimizing undesired off-target effects.

In specific instances, GO formulations functionalized using follicle-stimulating hormone receptor monoclonal antibodies have displayed enhanced specificity and doxorubicin delivery to cancer cells in a metastatic breast cancer model animal.^84^ Similar to these cancer models, GOs can be functionalized with ligands such as CD98 antibody,^85^ peptide transporter 1 (PepT1),^86^ and cyclin D1^87^ to enhance the targeting of intestinal epithelial and immune cells in IBD.

While the Lgr5^+ve^ intestinal organoids replicate the 3D intestinal structure in vitro, they are not without limitations. One major limitation of the intestinal organoid system used here is the lack of functioning mesenchyme and immune cells such as macrophages. Dysregulation of immune cells is one of the primary causes of IBD.^88^ Mesenchymal cells are responsible for the deposition of extracellular matrix (ECM).^89^ In IBD, inflammatory factors produced by macrophages can dysregulate the production of ECM-degrading MMPs and MMP inhibitors.^89^ This can severely impair intestinal homeostasis and disrupt the mucosal barrier. Thus, an in vitro model of IBD that incorporates a functional mesenchyme and immune component would be a more biologically relevant model for the testing of nanotherapeutics, a goal we are continuously working on.^88^

## Conclusions

Intestinal organoids, specifically enteroids and colonoids, manifest as ex vivo self-organizing cellular structures closely recapitulating the structural and functional characteristics of their in vivo counterparts. These organoids serve as an exceptional biomimetic predictive platform for disease modeling and the assessment of drug and biologic therapeutic effectiveness.^90^ In our study, mouse enteroids and colonoids were cultured to exhibit an organotypic morphology, featuring well-defined basal and luminal compartments. These organoids were subjected to prolonged exposure to inflammatory factors, resulting in the development of an inflammatory phenotype similar to that of IBD. Subsequently, these organoid models were leveraged to evaluate the potential of graphene oxide-mediated transfection of TNF-α_siRNA for mitigating the inflammatory response in IBD. Three different formulations of GOs were employed as carriers for delivering TNF-α_siRNA into inflamed organoids. Among these formulations, PEG and dendrimer-modified small GO (smGO) showed the highest transfection efficiency, which was attributed to its small flake size and chemical modifications. This improved transfection efficiency was found to be correlated with the downregulation of TNF-α and associated cytokines. Additionally, improvements in the organoid morphology and a reduction in apoptosis were observed. Furthermore, augmenting the TNF-α_siRNA/smGO ratio from 1:1 to 3:1 further enhanced the transfection efficiency and the attenuation of the inflammatory phenotype. Taken together, these findings underscore the significance of utilizing tissue-specific organoids with disease-relevant phenotypes as a valuable ex vivo model for assessing the therapeutic efficacy of nanocarrier-mediated drug and biologic delivery systems.

## Supporting Information Available

Additional TLR4 and TNFR1 tests, morphological scoring after treatment with inflammatory factors, and TNF-α_siRNA_smGO transfections (PDF)
