# Supplementary material for: Attenuation of Chronic Inflammation in Intestinal Organoids with Graphene Oxide-Mediated Tumor Necrosis Factor-α_Small Interfering RNA Delivery
Source: Langmuir. 2024 Feb 7;40(7):3402–13. doi: 10.1021/acs.langmuir.3c02741 (PMC10883062; doi:10.1021/acs.langmuir.3c02741)
Supplement: Supplementary file 1 — la3c02741_si_001.pdf [file la3c02741_si_001.pdf]

Supporting Information

Attenuation of Chronic Inflammation in Intestinal Organoids with Graphene Oxide-Mediated Tumor Necrosis Factor-α_small interfering RNA Delivery

*Sadman Sakib and Shan Zou**

Metrology Research Centre, National Research Council of Canada, 100 Sussex Drive, Ottawa, ON K1A 0R6, Canada

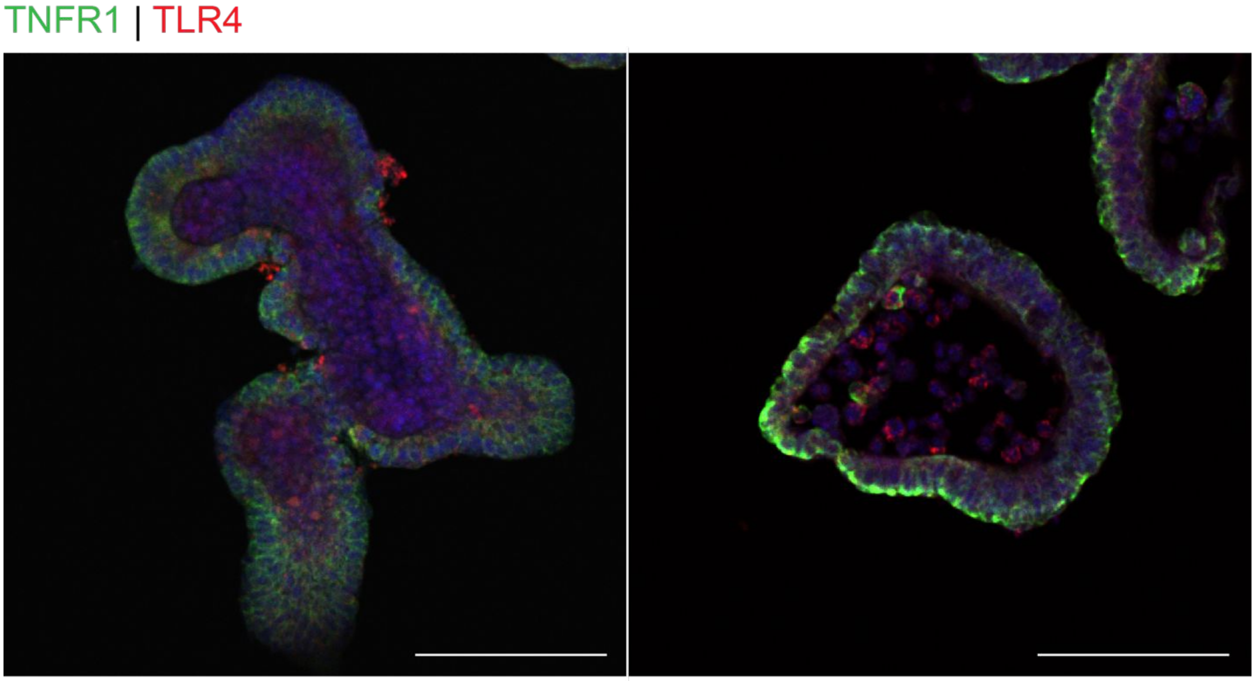

Figure S1. Intestinal organoids express mediators of inflammation. Immunofluorescence images of day 10 enteroids (left) and colonoids (right) showing TNFR1 (green), TLR4 (red) and nucleus (Hoechst, blue). Scale bars measure 500 μm.

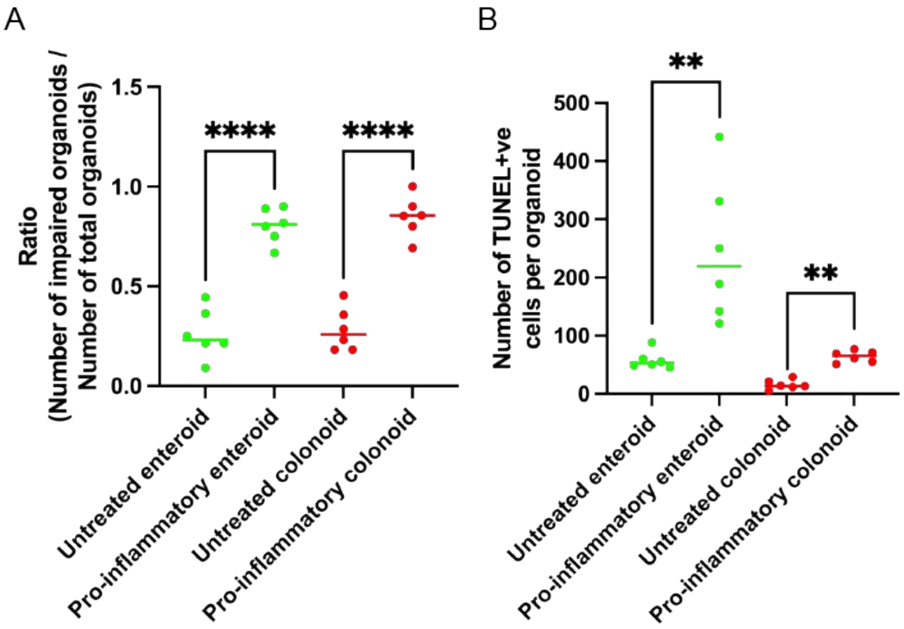

Figure S2. Treatments with inflammatory factors show impaired organoid morphology and increased apoptosis. (A). Morphological scoring of untreated organoids and organoids treated with pro-inflammatory factors. (B) Quantification of TUNEL^{+ve} cells in each organoid. Values on each graph are shown as mean ± SD of six independent experiments (n=6). Statistical significance was determined with t-tests conducted separately for enteroids and colonoids. P>0.05 was considered not significant. *P < 0.05; **P < 0.01; ***P < 0.001; and ****P < 0.0001.

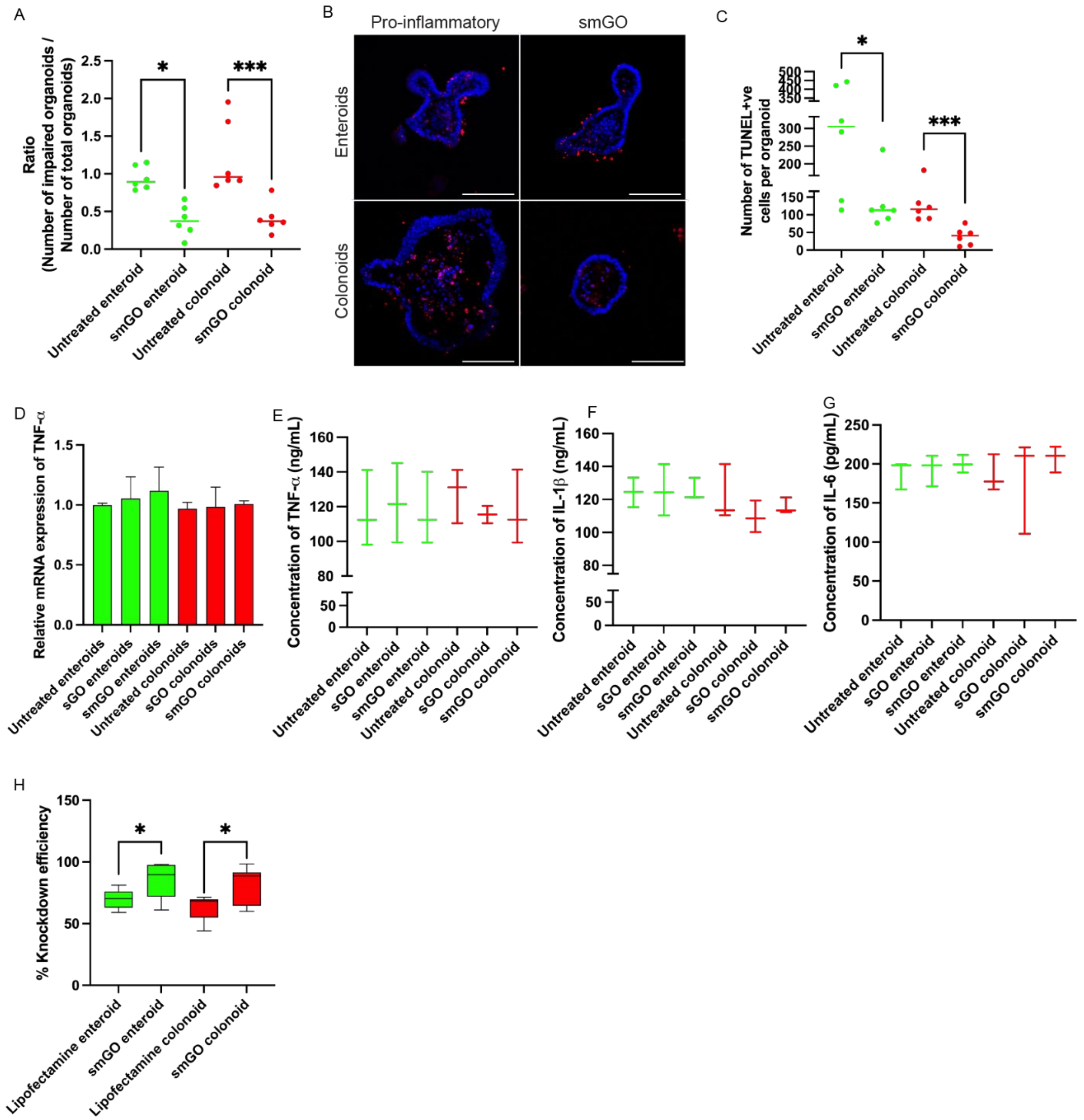

Figure S3. (A) Morphological scoring of untreated inflammatory organoids and inflammatory organoids treated with TNF-α_siRNA_smGO. (B) Representative TUNEL image of treated and untreated pro-inflammatory organoids. TUNEL^{+ve} cells are shown in red and nuclei are shown in blue. Scale bars measure 500 μm. (C) Quantification of TUNEL^{+ve} cells in each organoid. (D)

Relative mRNA expression of TNF-α in untreated organoids and organoids treated with sGO and smGO alone. No observable difference in protein concentrations for TNF-α (E), IL-1β (F) and IL-6 (G) in organoids treated with sGO and smGO alone. (H) Knockdown efficiency for TNF-α after transfections of enteroids and colonoids with Lipofectamine_TNF-α siRNA. Values on each graph are shown as mean ± SD of six independent experiments (n=6). Statistical significance was determined with a t-test (for comparing two groups in A, C and H) and one-way ANOVA (for comparing three groups or more in D-G). P>0.05 was considered not significant. *P < 0.05; **P < 0.01; ***P < 0.001; and ****P < 0.0001.
